# Green Leaf Volatile Emissions during High Temperature and Drought Stress in a Central Amazon Rainforest

**DOI:** 10.3390/plants4030678

**Published:** 2015-09-15

**Authors:** Kolby J. Jardine, Jeffrey Q. Chambers, Jennifer Holm, Angela B. Jardine, Clarissa G. Fontes, Raquel F. Zorzanelli, Kimberly T. Meyers, Vinicius Fernadez de Souza, Sabrina Garcia, Bruno O. Gimenez, Luani R. de O. Piva, Niro Higuchi, Paulo Artaxo, Scot Martin, Antônio O. Manzi

**Affiliations:** 1 Climate Science Department, Earth Science Division, Lawrence Berkeley National Laboratory, One Cyclotron Rd, building 74, Berkeley, CA 94720, USA; E-Mails: jchambers@lbl.gov (J.Q.C.); jaholm@lbl.gov (J.H.); 2 Department of Geography, University of California Berkeley, 507 McCone Hall #4740, Berkeley, CA 94720, USA; E-Mail: clafontes@gmail.com; 3 National Institute for Amazon Research (INPA), Ave. Andre Araujo 2936, Campus II, Building LBA, Manaus, AM 69.080-97, Brazil; E-Mails: angela.jardine@inpa.gov.br (A.B.J.); lotraquelu@gmail.com (R.F.Z.); viniciusfernandes11@yahoo.com.br (V.F.S.); sabrinagarcia.sg@gmail.com (S.G.); bruno.oliva.gimenez@gmail.com (B.O.G.); luanipiva@yahoo.com.br (L.R.O.P.); niro@inpa.gov.br (N.H.); aomanzi@gmail.com (A.O.M.); 4 Department of Neurobiology, The Barrow Neurological Institute, Saint Joseph’s Hospital and Medical Center, 350 W Thomas Rd, Phoenix, AZ 85013, USA; E-Mail: kmeyers1@email.arizona.edu; 5 Instituto de Fisica, Universidade de Sao Paulo, Rua do Matao, Travessa R, 187 Sao Paulo SP 05508-900, Brazil; E-Mail: artaxo@if.usp.br; 6 School of Engineering and Applied Sciences, Harvard University, Cambridge, MA 02138, USA; E-Mail: smartin@seas.harvard.edu; 7 Department of Earth and Planetary Sciences, Harvard University, Cambridge, MA 02138, USA

**Keywords:** leaf temperatures, drought, tree mortality, abiotic stress, green leaf volatiles

## Abstract

Prolonged drought stress combined with high leaf temperatures can induce programmed leaf senescence involving lipid peroxidation, and the loss of net carbon assimilation during early stages of tree mortality. Periodic droughts are known to induce widespread tree mortality in the Amazon rainforest, but little is known about the role of lipid peroxidation during drought-induced leaf senescence. In this study, we present observations of green leaf volatile (GLV) emissions during membrane peroxidation processes associated with the combined effects of high leaf temperatures and drought-induced leaf senescence from individual detached leaves and a rainforest ecosystem in the central Amazon. Temperature-dependent leaf emissions of volatile terpenoids were observed during the morning, and together with transpiration and net photosynthesis, showed a post-midday depression. This post-midday depression was associated with a stimulation of C_5_ and C_6_ GLV emissions, which continued to increase throughout the late afternoon in a temperature-independent fashion. During the 2010 drought in the Amazon Basin, which resulted in widespread tree mortality, green leaf volatile emissions (C_6_ GLVs) were observed to build up within the forest canopy atmosphere, likely associated with high leaf temperatures and enhanced drought-induced leaf senescence processes. The results suggest that observations of GLVs in the tropical boundary layer could be used as a chemical sensor of reduced ecosystem productivity associated with drought stress.

## 1. Introduction

As the single largest intact tropical forest in the world, the Amazon is a key component of the global carbon cycle. While a long-term trend as a net carbon sink has been observed, a high sensitivity to drought exists [1] and recent analysis of biomass dynamics revealed a long-term increasing trend of mortality-driven shortening of carbon residence times [2]. Thus, increased tree mortality reduces the ability of tropical forests to mitigate climate change by maintaining a net carbon sink. During the 2010 dry season, the Amazon Basin suffered a large-scale drought which resulted in widespread tree mortality and an estimated 2.2 × 10^15^ g of carbon lost from the forest to the atmosphere [3].

The ability of Amazon trees to withstand drought and high temperatures may partially be related to their capacity to counteract the buildup of reactive oxygen species (ROS) under abiotic stress. Plants mitigate the impacts of abiotic stress via antioxidant and ROS quenching systems, which reduce the accumulation of ROS in plant tissues [4]. However, when ROS accumulation is excessive, extensive oxidation of sensitive lipid components can occur including membrane fatty acids and photosynthetic pigments, which support the light reactions of photosynthesis [5,6]. Thus, the over-production of ROS, including singlet oxygen, superoxide anion, hydrogen peroxide, and the hydroxyl radical, can result in the extensive peroxidation of the lipid components of membranes and the breakdown of carbon capture mechanisms [7]. Experimental evidence suggests that the biosynthesis of volatile terpenoids (isoprene, monoterpenes, and sesquiterpenes) in the leaves of many plant species plays an important role in energy consumption and antioxidant mechanisms which reduce the sources and increase the sinks for ROS, resulting in reductions in lipid peroxidation [8,9,10,11].

Although damage to important cellular structures including cellular membranes has traditionally been viewed as deleterious, ROS-lipid signaling is recognized as playing a key role in a large number of biological processes including plant response to abiotic and biotic stress as well the regulation of growth, development, and programmed cell death [12,13,14]. By functioning as reactive electrophile species [15], many lipid oxidation products serve as effective signaling compounds within and between plants that can initiate the expression of a wide array of defense genes [16]. Plant membranes, which support the light reactions of photosynthesis, are particularly sensitive to oxidative stress due to the high reactivity of unsaturated fatty acids towards radical initiated hydrogen abstraction and the subsequent formation of fatty acid hydroperoxides [17]. The degradation of fatty acid hydroperoxides formed via non-enzymatic [18,19] and enzymatic [20,21,22] mechanisms produces a broad range of oxidation product biomarkers termed oxylipins. Therefore, volatile oxylipins, which have sufficient vapor pressure to be emitted from leaves and other tissues as gases into the atmosphere under normal physiological conditions, can make up an important component of volatile emissions under drought and high temperature stress [6]. Following production in leaves, volatile oxylipins first must pass through the intercellular air space before entering the atmosphere via the stomata**.** In chloroplasts, type 2 lipoxygenase enzymes (13-LOX) initiate the peroxidation of plant fatty acids by catalyzing the oxygenation of α-linolenic acid to form 13-hydroperoxy linolenic acid (HPLA) [20]. Following the degredation of HPLA (by a hydroperoxide lyase), a number of volatile oxylipins are produced which are termed green leaf volatiles (GLVs), including C_6_ aldehydes, alcohols, and their corresponding acetate esters [23,24,25,26]. Under controlled laboratory studies, the emissions of GLVs from plants have been documented following a number of abotic and biotic stresses associated with ROS accumulation, including pathogen attack [27], high ambient ozone concentrations [22,28], herbivory [29], desiccation [30], high light and temperature [31], mechanical wounding [25], light-dark transitions [32], freeze-thaw events [33], and programmed cell death during senescence [34]. However, GLVs are rarely reported from field observations in natural ecosystems, particularly in the tropics during environmental extremes.

In this study, we hypothesized that during drought-induced leaf senescence in Amazon trees, the combined effect of high temperature and leaf desiccation can overwhelm antioxidant defense systems such as volatile terpenoids. These abiotic stress conditions can result in the peroxidation of photosynthetic membranes, a decrease in net carbon assimilation, and the emissions of GLVs to the tropical atmosphere. To test this hypothesis, we used proton transfer reaction-mass spectrometry (PTR-MS) to quantify high vertically resolved ambient concentrations of C_6_ GLVs within and above a primary rainforest canopy in the central Amazon rainforest during the widespread 2010 drought. In 2015, we also conducted diurnal water and temperature stress experiments with detached leaves at 20 m height at the Amazon field site. The results show that the combined effect of high leaf temperatures and water deficits can result in a strong decrease in photosynthesis, transpiration, and the emissions of volatile terpenoids with a simultaneous stimulation of GLVs. For the first time, we show that during the 2010 Amazon drought, GLVs accumulated within the rainforest canopy atmosphere, potentially providing a new chemical sensor of decreased ecosystem productivity.

## 2. Results and Discussion

**Figure 1:**
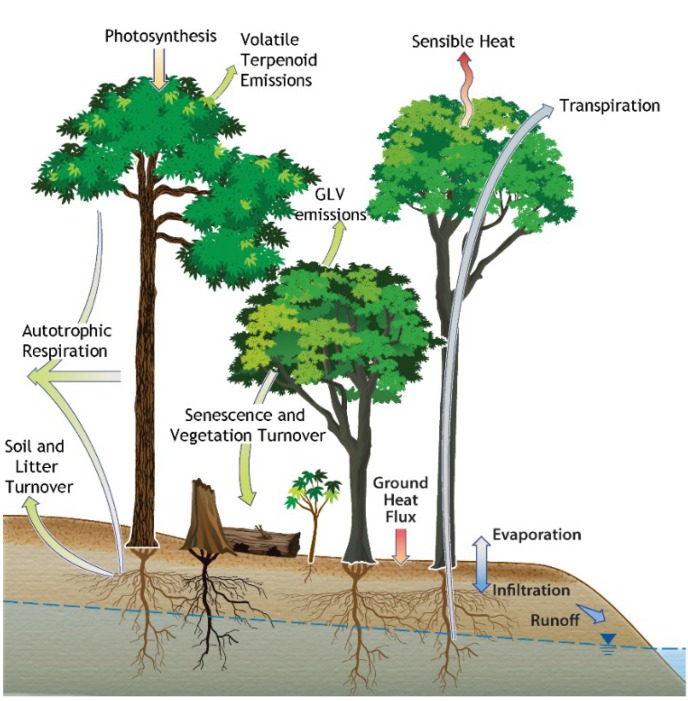
Simplified schematic of the interactions between the water and carbon cycle within the Amazon rainforest. Note the emissions of volatile terpenoids connected with photosynthesis and the emissions of GLVs connected with senescence and vegetation turnover.

In this study, we investigated the links between the carbon and water cycles in tropical forests during drought-induced leaf senescence and vegetation turnover (Figure 1). We evaluated the role of high leaf temperatures and low moisture availability on leaf senescence processes including membrane peroxidation and the emissions of GLVs at the leaf (2015) and ecosystem (2010) scales in the Amazon rainforest. At the Reserva Biológica do Cueiras (ZF2) in the central Amazon, we combined upper-canopy leaf detachment experiments under naturally varying conditions of light and temperature (K34 tower) with ecosystem-level ambient air studies (TT34 tower) to investigate possible foliar emissions of GLVs in response to drought and elevated leaf temperatures.

### 2.1. Leaf-Level Results

**Figure 2:**
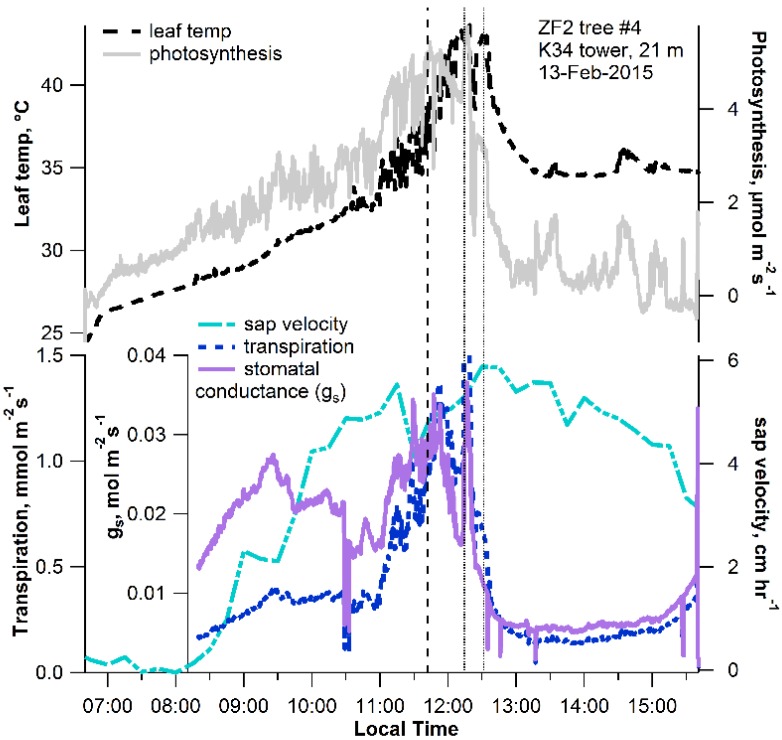
Example diurnal pattern of leaf temperature, net photosynthesis, transpiration, and stomatal conductance during leaf desiccation and high temperature stress following leaf detachment at 21 m on the K34 tower. Also shown is the associated diurnal pattern of sap velocity made from the *C. longipendula* tree trunk at the ZF2 forest reserve. Vertical dotted lines represent the peaks in net photosynthesis and transpiration (coarse dashed line) and peaks in leaf temperature (fine dashed lines).

**Figure 3:**
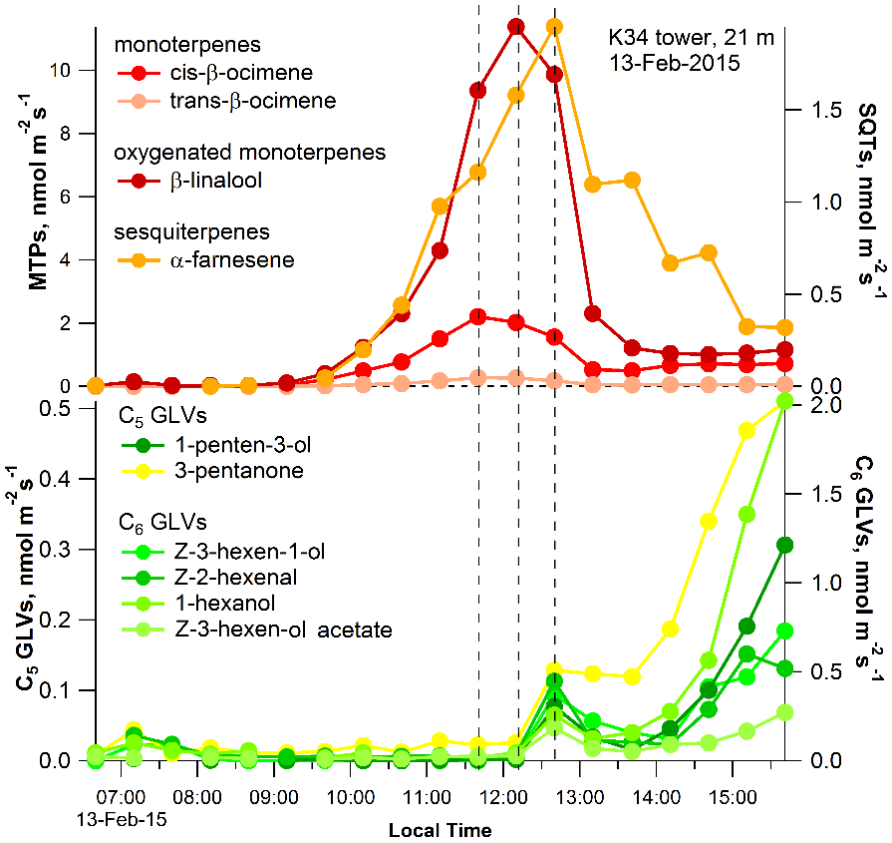
Example diurnal pattern of monoterpene, oxygenated monoterpene (MTP), sesquiterpene (SQT), and green leaf volatile emissions (C_5_ and C_6_ GLVs) under ambient conditions of light, temperature, and moisture, following leaf detachment at 21 m on the K34 tower. Note that during desiccation and high temperature stress of the *C. longipendula* leaf in the early afternoon, a strong decline in volatile terpenoid emissions is associated with a rise in GLV emissions. Vertical dotted lines represent the peaks in net photosynthesis and transpiration (coarse dashed line) and peaks in leaf temperature (fine dashed lines).

In order to examine the combined effect of leaf water deficit and high leaf temperatures on membrane peroxidation during the senescence process, individual leaves from a *Couepia longipendula* Pilg. tree at 21 m height near the K34 tower (Lat. 02°36′ S; Long. 60°12′ W) were detached (6 a.m.) and placed in the leaf enclosure for gas-exchange measurements, including volatile emissions, using thermal desorption GC-MS. Diurnal patterns of whole tree sap flux were also collected together with leaf gas-exchange fluxes of CO_2_, H_2_O, and volatile terpenoids under ambient light, temperature, and moisture conditions (Figure 2 and Figure 3). Trunk sap flux at 1.5 m height showed a strong diurnal pattern with a clear increase in the morning (8 a.m.) and reaching a maximum at 12:40 p.m. and declining thereafter. Upon receiving sunlight in the morning (~7 a.m.), transpiration, net photosynthesis, and stomatal conductance of the detached leaf increased together with leaf temperature up until 11:40–11:50 a.m., when temperatures reached 36–38 °C (Figure 2). In addition, temperature-dependent leaf emissions of a monoterpene (cis-β-ocimene), an oxygenated monoterpene (β-linalool), and a sesquiterpene (α-farnesence) peaked between 11:40 a.m. and 12:40 p.m., when the highest leaf temperatures (36–42 °C) occurred (Figure 3).

Thus, the peak in net photosynthesis and transpiration coincided or slightly preceded the peaks in volatile terpenoid emissions with respect to both time and leaf temperature. This may be due to the increased availability and use of photoassimilates and energy metabolites for monoterpene biosynthesis in chloroplasts via the methylerythritol phosphate pathway (MEP) [35], which may interact with the cytosolic mevalonic acid pathway for sesquiterpene biosynthesis through the exchange of common intermediates [36]. Several protective and ecological functions have been identified for volatile terpenoids during abiotic stress. Isoprene has been shown to increase the thermotolerance of photosynthesis [37], reduce membrane peroxidation [38], and quench ROS within plant tissues including ozone [39], hydrogen peroxide [38], and singlet oxygen [40]. Although this effect is less known for monoterpenes, they have also been shown to offer protection to net photosynthesis under stress, including high temperatures [41] and during ozone fumigation [42]. Recent observations in the tropics revealed the emission of highly reactive monoterpenes like cis- and trans-β-ocimene from several abundant species [35]. In contrast, much less is known about the role of sesquiterpenes in plants, although a number of studies reported induced emissions following abiotic stress such as mechanical damage [43].

At the highest leaf temperature (42 °C, 12:40 p.m.), a burst in both C_5_ and C_6_ GLV emissions occurred followed by a decline until 13:30, when leaf temperatures decreased to 35 °C. Throughout the rest of the experiment (1:30 p.m.–3:30 p.m.), a gradual decrease in volatile terpenoid emissions occurred together with a stimulation in C_5_ and C_6_ GLVs emissions, which continued to increase throughout the afternoon despite the stabilization in leaf temperatures at around 35 °C. The afternoon (1:30 to 3:30) was therefore characterized by low rates of net photosynthesis, stomatal conductance, transpiration, and emissions of volatile terpenoids. In contrast, high leaf emission rates of GLVs were observed in an apparent non-temperature-dependent fashion. Therefore, in the Amazon study at the leaf level, a morning temperature-dependent increase in net photosynthesis and volatile terpenoid emissions was observed and replaced by temperature-independent emissions of GLVs in the afternoon. These observations suggest that the collapse of photosynthesis and the volatile isoprenoid antioxidant system in the afternoon under high temperature and desiccation stress is associated with membrane peroxidation and the emissions of GLVs.

### 2.2. Ecosystem-Level Results

**Figure 4:**
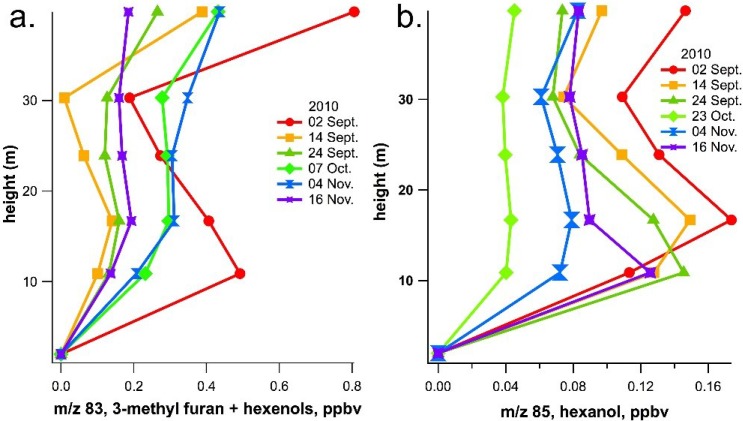
Mean daytime vertical profiles of GLVs as estimated by PTR-MS on (**a**) *m*/*z* 83 (3-methyl furan + hexenols) and (**b**) *m*/*z* 85 (hexanol) within and above a 30 m rainforest canopy in the central Amazon. Each vertical profile represents the daytime (10 a.m.–4 p.m.) average of continuous observations over a three- to five-day period (starting date shown in legend). Note the buildup of GLVs within the canopy during the dry season as well as a possible contribution of 3-methyl furan from isoprene oxidation above the canopy. The standard deviation at each height was between 20% and 40% of the mean GLV concentration values.

**Figure 5:**
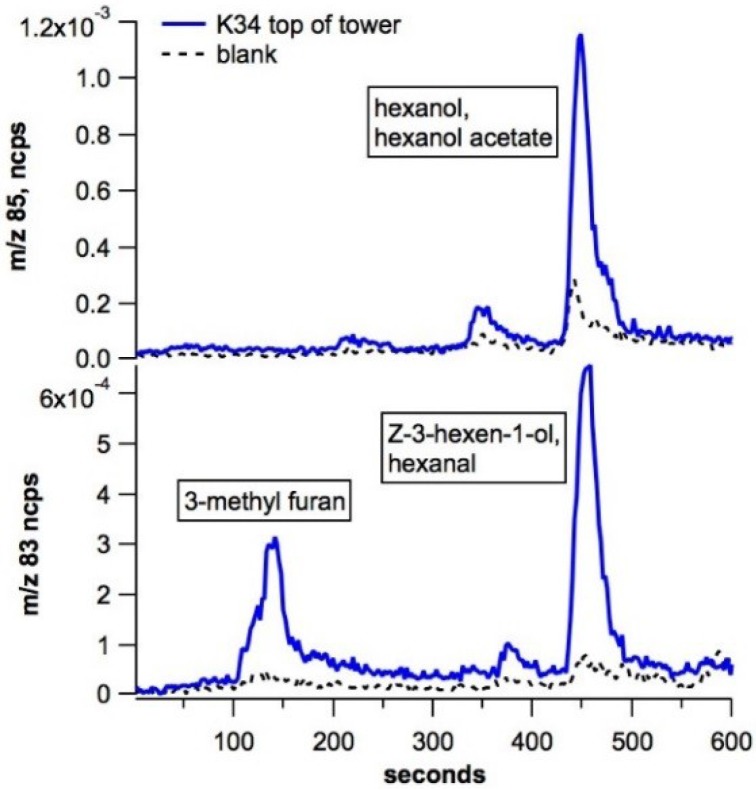
Example GC-PTR-MS selected ion chromatogram of an air sample collected above the central Amazon forest canopy (top of K34 tower, 50 m) during the 2010 drought showing the presence of C_6_ GLVs. Note the presence of both 3-methyl furan and Z-3-hexen-1-ol on the PTR-MS signal at *m*/*z* 83.

Atmospheric vertical profiles of GLVs were measured using PTR-MS on the TT34 tower within and above the 30 m rainforest canopy during the 2010 dry season. Monthly resolved daytime vertical profiles of C_6_ GLVs revealed that September, the warmest month, generally showed the highest ambient GLV concentrations (Figure 4). Moreover, 3-methyl furan + hexenols showed higher concentrations (up to 0.8 ppb) than hexanol (up to 0.2 ppb) (Figure 4). For both 3-methyl furan + hexenols (*m*/*z* 83) and hexanol (*m*/*z* 85), a buildup in ambient concentrations was observed within the canopy between 11 and 17 m. This is the same region of the canopy where isoprene concentrations reach a maximum, suggesting a canopy source for GLVs. However, an elevated signal for 3-methyl furan + hexenols (*m*/*z* 83) was also detected at 40 m (10 m above the 30 m canopy). Thermal desorption GC-PTR-MS analysis of ambient air collected above the tower was used to discriminate between potential contributions from 3-methyl furan and hexenols on the PTR-MS signal at *m*/*z* 83 (Figure 5). GC-PTR-MS chromatograms showed that the ambient *m*/*z* 85 signal is largely due to a single compound (hexanol), with a retention time of roughly 450 s (7.5 min). In contrast, GC-PTR-MS chromatograms reveal two peaks for the *m*/*z* 83 signal occurring at 140 s (2.3 min) and 460 s (7.7 min), likely corresponding to 3-methyl furan (an oxidation product of isoprene) and hexenols, respectively. The peak in the *m*/*z* 83 signal both within and above the 30 m canopy may be due to a combination of sources, including direct emissions from vegetation, transport from nearby local vegetation sources above the canopy, and/or the formation of 3-methyl furan from isoprene photooxidation above the canopy.

## 3. Experimental Section

### 3.1. Site Description

The study was carried out at the Reserva Biológica do Cueiras in central Amazonia, 60 km northwest of the city of Manaus, Brazil. The site, also known as ZF2, is run by INPA (Instituto Nacional de Pesquisas da Amazonia) under the Large Scale Biosphere-Atmosphere Experiment in Amazonia (LBA) program [44]. The vegetation in this area is considered to be undisturbed, mature, *terra firme* rainforest, with a leaf area index of 5–6 and an average canopy height of 30 m [45].

### 3.2. Leaf Level Desiccation Study Following Leaf-Detachment from Tree

In order to study the combined effects of leaf desiccation and high temperatures, in February 2015, diurnal patterns of leaf volatile emissions together with net photosynthesis rates, stomatal conductance, and transpiration rates were collected following leaf detachment in the Reserva Biológica do Cueiras (ZF2). Two sun-lit upper canopy leaves from a tree (*Couepia longipendula* Pilg.) accessible from the K34 walkup tower at a height of roughly 20 m were detached between 5 a.m. and 6 a.m. and placed in a portable photosynthesis system mounted on the K34 tower (20 m) and coupled to a volatile auto-sampler system (Less-P, Signature Science, Inc., Austin, TX, USA). This analytical configuration has been described previously [35], but with leaf temperature and photosynthetically active radiation (PAR) allowed to vary naturally throughout the day using the standard leaf chamber top. Volatiles in the leaf enclosure headspace were sequentially collected every hour on inert coated stainless steel thermal desorption (TD) tubes purchased commercially, filled with Quartz wool, Tenax TA, and Carbograph 5TD adsorbents (Markes International, Inc., Cincinnati, OH, USA) at a flow rate of 100 mL/min for 60 min (6.0 L). Thermal desorption tubes were analyzed for volatile terpenoids and GLVs using a thermal desorption system (TD-100, Markes International) interfaced with a gas chromatograph/electron impact mass spectrometer with a triple-axis detector (5975C series, Agilent Technologies, Santa Clara, CA, USA). The GC-MS was calibrated to authentic volatile terpenoids and GLV standards (99%, Sigma Aldrich, St. Louis, MO, USA) in methanol using the dynamic solution injection (DSI) technique [46]. Sap flow of the *C. longipendula* tree was continuously measured in 15 min intervals throughout the leaf-detachment experiments using a commercial sap flow meter installed in January 2015 (SFM1, ICT International, Armidale, Australia).

### 3.3. Ambient Air Studies

Atmospheric gradients of GLVs were collected on the TT34 tower using six ambient air inlets at different tower heights (2, 11, 17, 24, 30, and 40 m) sequentially analyzed for C_6_ GLVs by proton transfer reaction-mass spectrometer (PTR-MS) for 10 min at each inlet (one complete canopy profile per hour). Ambient air was drawn through one-quarter in O.D. Teflon PFA tubing using an oil-free diaphragm pump (KNF Neuberger) with a sample point to detector delay time of <15 s. Prior to each vertical gradient ambient air measurement period (lasting 4–7 days), ultra-high purity nitrogen was run for two hours directly into the PTR-MS to obtain instrument background signals. Vertical gradients were calculated by averaging the last seven minutes of each 10-minute sampling period. Average vertical gradients during the day (10 a.m.–4 p.m.) were calculated for each measurement period during the 2010 dry season occurring between 2 September 2010 and 20 November 2010.

Ambient concentrations of C_6_ GLVs were quantified using a commercial high sensitivity proton transfer reaction-mass spectrometer (PTR-MS, Ionicon Inc., Innsbruck, Austria). The PTR-MS was operated under standard conditions with a drift tube voltage of 600 V and drift tube pressure of 2.0 mb. Optimization of PTR-MS conditions resulted in extremely high and sustained primary ion intensities (20–40 MHz H_3_O^+^) with low water cluster (H_2_O-H_3_O^+^ < 4% H_3_O^+^) and O_2_^+^ (O_2_^+^ < 4% H_3_O^+^) formation. The following mass-to-charge ratios (*m*/*z*) were sequentially monitored during each PTR-MS measurement cycle; 21 (H_3_^18^O^+^), 32 (O_2_^+^), 37 (H_2_O-H_3_O^+^) with a dwell time of 20 ms each, and 83 (3-methyl furan + hexenols) and 85 (hexanol) with a dwell time of 5 s each [25]. The PTR-MS was calibrated to GLVs using the dynamic solution injection technique at the beginning of the field campaign. The 0–4 μL·min^−1^ of a calibration solution (0.3 mM Z-3-hexen-1-ol and hexanol in cyclohexane) was evaporated into a 1.0 L·min^−1^ hydrocarbon-free air dilution stream and analyzed by the PTR-MS. GC-PTR-MS analysis of GLVs in ambient air was based on thermal desorption as previously described [47]. Briefly, above canopy air samples (100 mL/min × 15 min) were collected at 50 m height on thermal desorption tubes packed with Tenax-TA and carbograph 5TD sorbents and analyzed for GLVs by desorbing at 200 °C in helium carrier gas directly onto the analytical column (Rtx-volatiles, Restek, 30 m × 0.53 mm × 2 μm) held under isothermal conditions (40 °C). Compounds eluding from the analytical column were immediately directed into the PTR-MS inlet.

## 4. Conclusions

In this study, high vertically resolved atmospheric concentrations of GLVs were collected within and above a rainforest ecosystem in the central Amazon during the 2010 dry season. Throughout this period (June–September 2010), widespread drought conditions were reported for the Amazon rainforest, and together with high heat anomalies, resulted in large reductions in above-ground biomass [48]. For the first time, we observed elevated ambient concentrations and ecosystem emissions of C_6_ GLVs, potentially in response to the widespread 2010 drought, as implied from the buildup of ambient GLV concentrations within the canopy. Additional experiments at the leaf-level in 2015 at the Amazon field site verified that emissions of both C_5_ and C_6_ GLVs can be induced by high temperature and desiccation stress and are associated with the loss of net photosynthesis, transpiration, and volatile terpenoid emissions, likely due to the peroxidation of photosynthetic membranes. Drought intensity and duration is expected to increase in some regions of the Amazon in upcoming decades with a potentially large impact on the pantropical terrestrial carbon sink [49]. Our observations suggest that during the drought-induced leaf senescence processes, volatile terpenoid emissions may initially be stimulated but decline following photosynthetic membrane peroxidation and the loss of net carbon assimilation. Thus, if volatile terpenoids act as effective antioxidants to reduce photosynthetic membrane peroxidation during stress, this antioxidant system can be overwhelmed and/or is no longer active during excessive ROS accumulation associated with leaf senescence, lipid peroxidation, and the emissions of GLVs to the atmosphere. We conclude that observations of GLV emissions from tropical rainforests like the Amazon may provide a better understanding of the biological and environmental factors that reduce the ability of tropical rainforests to maintain net carbon uptake.
